# Calorimetry as a tool to improve the dosimetric accuracy in novel radiotherapy modalities

**DOI:** 10.1016/j.phro.2023.100516

**Published:** 2023-11-14

**Authors:** Felix Horst

**Affiliations:** a OncoRay – National Center for Radiation Research in Oncology, Faculty of Medicine and University Hospital Carl Gustav Carus, Technische Universität Dresden, Helmholtz-Zentrum Dresden – Rossendorf, Dresden, Germany; b Helmholtz-Zentrum Dresden – Rossendorf, Institute of Radiooncology - OncoRay, Dresden, Germany

The main quantity of interest in radiotherapy dosimetry is absorbed dose to water, i.e. the energy that is deposited by the radiotherapy beam in water per unit mass. The most common method to measure dose in radiotherapy is by using air-filled ionization chambers via the charge released in their active volume by ionizations. These ionization chambers are typically absolute calibrated in ^60^Co beams in terms of absorbed dose to water. If a measurement is carried out in another radiation quality (e.g. proton beams), the different response of the chamber in that radiation quality compared to ^60^Co photons due to a different water-to-air stopping power ratio, a different amount of electron-ion pairs produced in air per deposited energy and chamber-specific geometry effects is taken into account by applying a beam quality correction factor k_Q_. Because k_Q_ might be sensitive to several factors, it is recommended that absolute absorbed dose to water measurements should be performed within so-called reference conditions (e.g. defined field size and water depth), and therefore such measurements are referred to as reference dosimetry [1]. In addition to k_Q_, also several other corrections may be necessary (e.g., recombination or air density correction).

Compared to ionization chamber dosimetry, a more direct way to measure dose is calorimetry where the deposited energy in the detector is measured via its temperature increase. Calorimetry is considered as the most accurate method of dose determination but requires a large logistic effort, stable thermal conditions in the room plus a good insulation and those devices are usually very sensitive and complicated to operate. Therefore calorimetry is at present mostly applied as primary standard for absorbed dose in permanently installed setups at national metrology institutes [2], to which the calibration of ionization chambers used in radiotherapy clinics can be traced back to.

The natural choice of the calorimeter medium is water because absorbed dose to water is the quantity of interest in radiotherapy dosimetry. Due to some practical limitations of water calorimeters, there are also calorimeter designs based on solid materials, typically graphite. Graphite calorimeters can be a lot more compact than water calorimeters and due to the smaller specific heat capacity of graphite, the temperature increase (i.e., the measurement signal) is about a factor five higher than for water at the same dose. However, the higher thermal conductivity of graphite requires additional insulation of the calorimeter core. Another characteristic of graphite calorimetry is that it requires a conversion from absorbed dose to graphite to absorbed dose to water and therefore the stopping power ratio in the radiation field of interest must be calculated.

Besides applications as primary standard for absorbed dose to water, calorimetric measurements can also be helpful to guarantee the dosimetric accuracy when novel radiotherapy modalities, for which standard dosimetry protocols are not suitable, are introduced. Recent examples are magnetic resonance guided radiotherapy [3], where the response of ionization chambers is modified by the magnetic field, or FLASH radiotherapy at ultra-high dose rate (UHDR) [4], [5] where recombination effects in ionization chambers become more pronounced than in conventional radiotherapy. For calorimetric measurements, the UHDR delivery can even be considered an advantage because the quasi-instantaneous dose application makes the heat drift become less relevant. For instance at the Physikalisch-Technische Bundesanstalt (PTB) in Germany, efforts were made to establish a water calorimeter as primary standard in the UHDR beam of their 20 MeV electron accelerator [4]. Another example is the first proton FLASH patient trial at the Cincinnati Children’s Hospital Medical Center in the USA where a group from the National Physical Laboratory (NPL) of the United Kingdom supported the dosimetric characterization of UHDR beams with their graphite calorimeter [5]. Recently, water calorimeters have been used to determine ionization chamber specific beam quality correction factors in clinical proton [6] and carbon ion beams [7], [8].

Generally, for protons and heavy ions no actual primary standards have been established up to now [9], because the national metrology institutes do not have suitable accelerators and beam qualities on-site but would have to travel to clinical facilities with their calorimetry equipment. For this purpose, since several years many metrology groups work on the development of portable calorimeters (see for example ref. [10] for an early work). At NPL a portable graphite calorimeter was developed [11]. This device is now intended to be applied for secondary standard measurements in UHDR proton beams in order to improve the dosimetric accuracy for this novel radiotherapy modality. Like ionization chamber dosimetry, also calorimetry requires a number of correction factors to be applied to the measured signal. In the present ESTRO 2023 conference physics highlight special issue, Cotterill and colleagues present detailed Monte Carlo simulations for their so-called Small-body Portable Graphite Calorimeter [12]. They derived correction factors for 250 MeV protons correcting for the graphite impurity and the air gap between the graphite core and its jacket. They show that the dominating perturbation (almost 0.5 %) is due to missing scatter contributions from the styrofoam insulation around the device, for which they introduce a new correction factor. By applying the obtained correction factors, the dosimetric accuracy of the calorimeter can be improved considerably. The in- and out-scattering of protons from the different components of the device was studied in detail and the dose conversion factor from absorbed dose in the graphite core to absorbed dose to water at the reference point was calculated.

Even though portable calorimeters like the one presented by Cotterill et al. are still more complicated to operate than ionization chambers, they are much more convenient to transport and set up than classic calorimetry setups. It will be very interesting to see if these developments will contribute to a wider spread of calorimetric measurements in radiotherapy, or even a routine use in radiotherapy clinics as envisioned by Cotterill et al., and if the establishment of a primary standard for absorbed dose to water in proton (and heavy ion) beams will finally succeed.

## Declaration of Competing Interest

The author declares that they have no known competing financial interests or personal relationships that could have appeared to influence the work reported in this paper.
